# Synthesis and Insecticidal Activity of Mesoionic Pyrido[1,2-α]pyrimidinone Derivatives Containing a Neonicotinoid Moiety

**DOI:** 10.3390/molecules23051217

**Published:** 2018-05-19

**Authors:** Jianke Pan, Lu Yu, Dengyue Liu, Deyu Hu

**Affiliations:** State Key Laboratory Breeding Base of Green Pesticide and Agricultural Bioengineering, Key Laboratory of Green Pesticide and Agricultural Bioengineering, Ministry of Education, Guizhou University, Huaxi District, Guiyang 550025, China; jkpan_1@163.com (J.P.); yuji570@163.com (L.Y.); 18230826058@163.com (D.L.)

**Keywords:** mesoionic, pyrido[1,2-α]pyrimidinones, neonicotinoid, insecticidal activity, proteomic

## Abstract

Mesoionic pyrido[1,2-α]pyrimidinone derivatives containing a neonicotinoid moiety were designed, synthesized, and evaluated for their insecticidal activity. Some of the title compounds showed remarkable insecticidal properties against *Aphis craccivora*. Compound **I13** exhibited satisfactory insecticidal activity against *A. craccivora*. Meanwhile, label-free proteomics analysis of compound **I13** treatment identified a total of 821 proteins. Of these, 35 proteins were up-regulated, whereas 108 proteins were down-regulated. Differential expressions of these proteins reflected a change in cellular structure and metabolism.

## 1. Introduction

**Figure 1:**
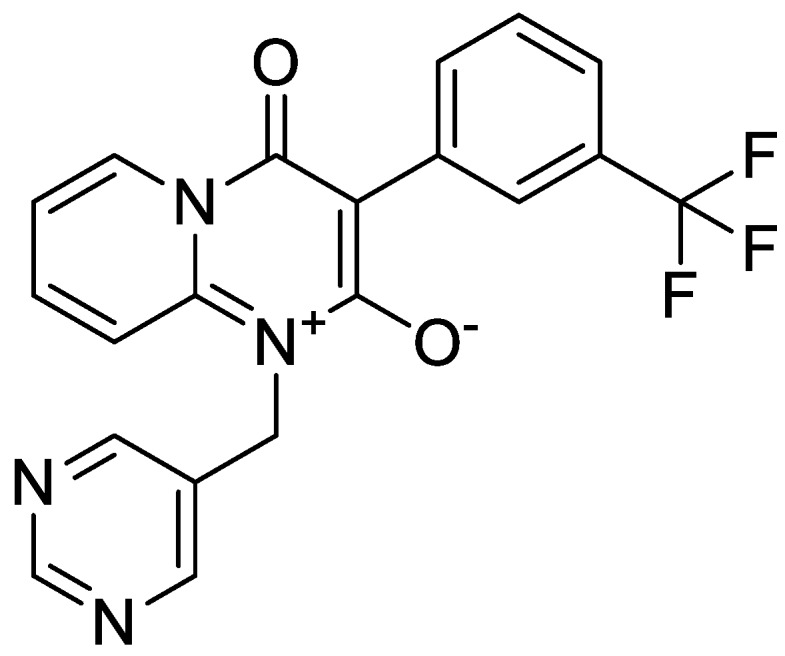
The structure of Triflumezopyrim registered in China in 2016.

Wide application of insecticides with the same mode of action has led to insect resistance; it is vitally important to develop novel insecticides with a new mode of action [1,2]. Mesoionic compounds are usually called non-benzenoid aromatics; these are polar and easily enter the hydrophilic cavity through the lipid barrier [3]. Besides these unique physical and chemical properties, they also possess various bioactivities, such as antifungal [4], anti-inflammatory [5,6,7], and analgesic activities [8,9,10,11]. They are inhibitors of cyclic AMP phosphodiesterase and antagonists of adenosine receptors [12,13], and show antibacterial [14,15,16], anti-tumor [17], and insecticidal [18,19,20,21,22] activities. Encouraged by these characteristics of mesoionic compounds, many researchers have studied the potential applications of mesoionics [23]. Recently, DuPont has discovered that Triflumezopyrim (Figure 1), a new commercial insecticide with a distinct mode of action and register in China in 2016, provides new insight into the application of mesoionic compounds in pesticides with high efficiency and environmentally friendly properties [19,24,25]. Mesoionic compounds in this field were also studied by DuPont [21]. Mesoionic compounds may be considered a novel pesticide.

Neonicotinoids are widely used to prevent and control various diseases in plants, animals, and humans [26]. They are the newest class of synthetic insecticides to emerge in the past two decades, and they are also the best-selling insecticide [27]. Many neonicotinoid pesticides have been launched to the market, including imidacloprid [28], nitenpyram [29], acetamiprid [30], thiamethoxam [31], clothianidin [32], and thiacloprid [33]. Additionally, 2-Cl-pyridin-5-yl and 2-Cl-thiazol-5-yl moieties play an important role in building the neonicotinoid insecticides; they are also the most insecticidal moieties of neonicotinoid insecticides.

**Figure 2:**
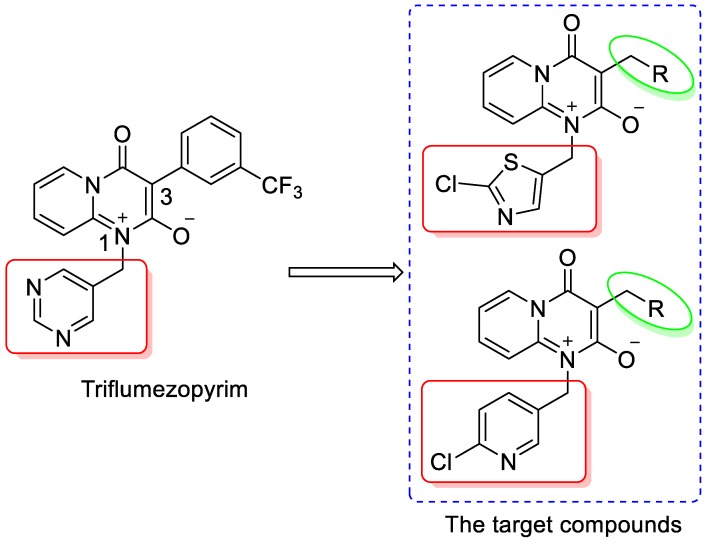
Design route of the target compounds.

As shown in Figure 2, we aimed to introduce a neonicotinoid moiety into 1-position of mesoionic pyrido[1,2-α]pyrimidinones and introduce a 2-Cl-pyridin-5-yl or 2-Cl-thiazol-5-yl moiety into 3-position of mesoionic pyrido[1,2-α]pyrimidinones to build some novel compounds. In this paper, we reported the synthesis and their insecticidal activity against *Aphis craccivora* of two series of mesoionic pyrido[1,2-α]pyrimidinone derivatives containing a neonicotinoid moiety. Moreover, the label-free proteomics technique was used to study the protein differences after compound **I13** treatment.

## 2. Results and Discussion

### 2.1. Chemistry

**Scheme 1:**
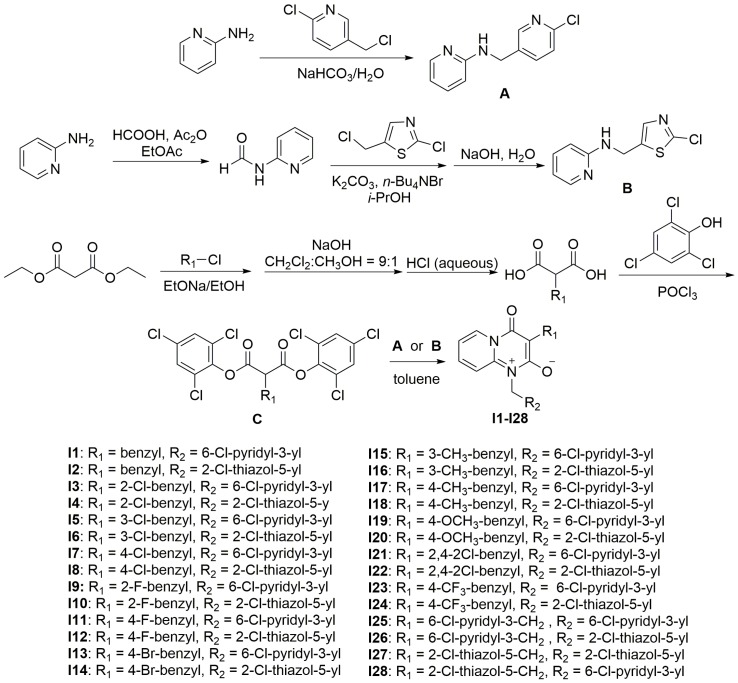
General synthetic procedure for the title compounds **I1**–**I28**.

As shown in Scheme 1, intermediates **A**, **B**, **C**, and the title compound **I** were prepared according to the reported methods. The structures of the title compounds were characterized by melting point, ^1^H-NMR, ^13^C-NMR, and HRMS. All copies of the spectrum for compounds **I1**–**I28** are available in Supplementary Materials.

### 2.2. Biological Evaluation

The insecticidal activities of the title compounds **I1**–**I28** against *A. craccivora* were assayed. The commercial agent imidacloprid and triflumezopyrim were used as controls. The biassay showed that the title compounds exhibited moderate activities. Among the title compounds, **I13** exhibited good insecticidal activities against *A. craccivora* with a mortality rate of 100% at 500 and 200 μg/mL, respectively, which was equal to those of imidacloprid (100%) and triflumezopyrim (100%). When the concentration of the compound **I13** is reduced from 200 to 100 μg/mL, compound **I13** still shows good mortality rate (92%) against *A. craccivora*. However, a great decrease of bioactivity (30%) was observed when the concentration was reduced from 100 to 50 μg/mL (Table 1). Meanwhile, at 500 μg/mL, compounds **I1**, **I2**, **I6**, **I7**, **I8**, **I19**, **I20**, **I22**, **I23**, **I25**, and **I28** exhibited moderate activities (85%, 69%, 51%, 77%, 51%, 58%, 62%, 62%, 58%, 62%, and 62%, respectively) against *A. craccivora*. However, other compounds exhibited weak and inactivitive activities. Based on the above findings, when R_2_ was 2-Cl-pyridin-5-yl group, the substituents of phenyl ring R_1_ on the parent compound **I** affected the activity against *A. craccivora*. In short, the position of the substituents is a key factor, while the electron effect of the substituent is a secondary factor. The target compounds having a *p*-position substituent exhibit good activities (**I7**, **I13**, **I19**, and **I23**), and the target compounds having an *o*-position substituent exhibit weak activities (**I3** and **I9**), while the target compounds having a *m*-position substituent show no activities (**I5** and **I15**). Meanwhile, **I25** and **I27** with heterocycle moiety exhibited moderate activity. When R_2_ turns to 2-Cl-thiazol-5-yl group, the compounds exhibit lower activity. Interestingly, when R_1_ was benzyl with no substituents on the benzene ring, the corresponding compounds showed moderate insecticidal properties, such as **I1** (mortality rate: 85%) and **I2** (mortality rate: 69%). In a word, the structures R_1_ and R_2_ of title compounds were combined with the activity. Among them, compound **I13** could offer considerable potential for further development as a new lead compound in modern drug discovery.

### 2.3. Label-Free proteomics Comparative Analysis

#### 2.3.1. Analysis of Protein between Control and Treatment Groups

**Figure 3:**
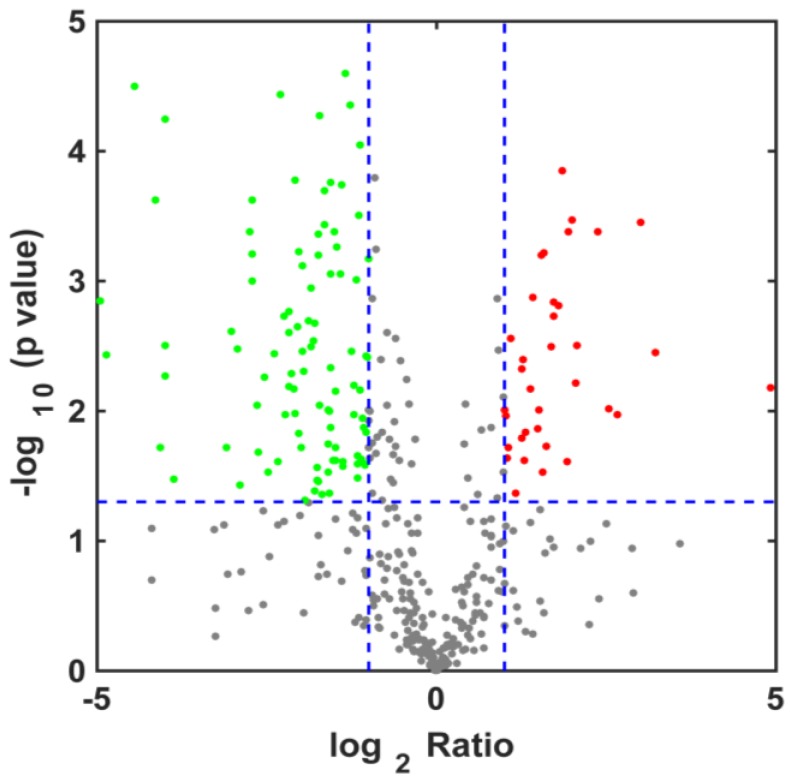
The up-regulated and down-regulated proteins in the control and treatment groups.

MaxQuant (version 1.5.2.8) search results identified 821 proteins, which were listed in Supplementary Materials (Supplementary Materials Table S1). As shown in Supplementary Materials Table S1, 678 proteins (82.6%) had non-specific expression, and 143 proteins were differentially expressed, out of which 35 proteins were up-regulated, whereas 108 proteins were down-regulated. Meanwhile, a volcanic map (Figure 3) was plotted to better understand the expression of this differential proteins (Supplementary Materials Table S2), which included 35 up-regulated proteins (red dots) and 108 down-regulated proteins (green dots).

#### 2.3.2. Bioinformatics Analysis

**Figure 4:**
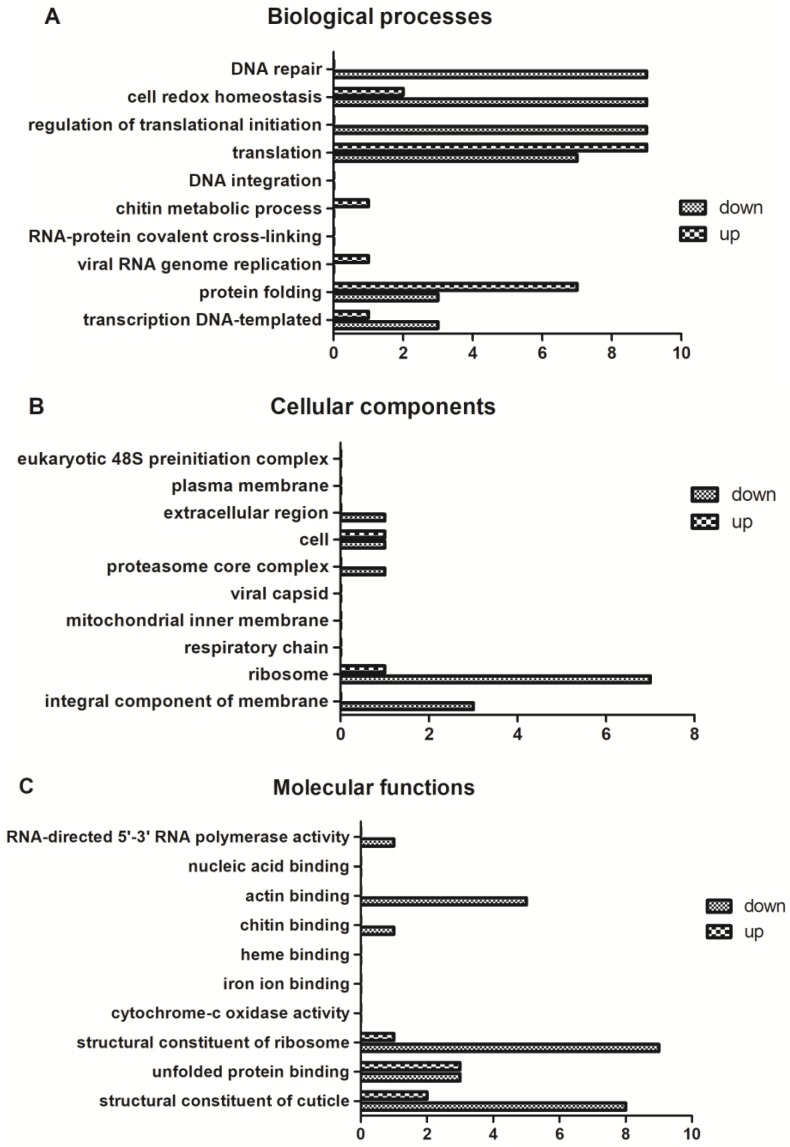
Some different expression proteins were grouped according to BP (**A**), CC (**B**), and MF (**C**).

Figure 4A shows the different expressions of proteins, which were grouped by biological process (BP). The up-regulated proteins were involved in protein folding and translation. The down-regulated proteins were mainly involved in DNA-templated transcription, protein folding, translation, regulation of translational initiation, cell redox homeostasis, and DNA repair. Grouped according to cellular components (CC), Figure 4B showed that integral component of membrane and ribosome showed decreased expression. The structural constituent of cuticle, structural constituent of ribosome, and actin binding were mapped to the up-regulated proteins of molecular function (MF) (Figure 4C).

To study the potential link between differentially expressed protein and biological functions, we used KEGG database to identify potential pathways for differential proteins in the treatment groups. Protein processing in endoplasmic reticulum (pathway ID: ko04141) is the main enrichment pathway. The enrichment pathway includes a total of 10 specific proteins, such as 6 heat shock proteins (HSP), 2 protein disulfide-isomerase, 1 transitional endoplasmic reticulum ATPase TER94, and 1 DnaJ-lik protein. HSP are in relation to temperature stress and a family of proteins that are produced by cells in response to exposure to stressful conditions. Furthermore, among different expressions proteins, we found some proteins were connected with temperature stress ((2 cold-shock proteins (IDS: Q492L6 and A0A0M3RSL4) and 2 HSP proteins (IDS: A0A172JCK4 and A0A0H5BX82)). Literatures revealed that CSPs can bind mRNA and regulate ribosomal translation, mRNA degradation, and the rate of transcription termination [33,34]. CSP, which can inhibit cell division and reducing apoptosis, is widely involved in the replication, transcription, translatin, protein folding, and membrane fluidity of various genes at low temperatures and plays a significant role in the protection of organ tissue at low temperatures [35,36,37]. However, the effect of cold shock had more of a general nature, e.g., slowing down of metabolic activities. Recent observations have changed this outlook on cold-shock response and have shown it to be a specific response of a cell at various levels, such as cytoplasmic membrane, ribosomes, nucleic acids, and proteins. So, we hypothesized that compound I13 can change the sensitivity to temperature and then lead to the death of *A. craccivora*.

## 4. Materials and Methods

### 4.1. Synthesis

NMR spectra were recorded on a JEOL ECX-500 spectrometer (JEOL, Tokyo, Japan). High-resolution mass spectra (HRMS) were acquired in positive mode on a MALDI SYNAPT G2 high-definition mass spectrometer (Waters, Milford, MA, USA). Melting points were taken on a Büchi B-545 melting point apparatus (Büchi Labortechnik AG, Flawil, Switzerland, uncorrected). Silica gel GF_254_-coated glass plates (Branch Qingdao Haiyang Chemical Co., Qingdao, China) were used for thin layer chromatography (TLC) under detection at 254 nm. Silica gel 200–300 mesh (Branch Qingdao Haiyang Chemical Co., Qingdao, China) was applied to column chromatography. All chemical reagents were commercially available and used without further purification.

Intermediates **A**, **B**, and **C** were prepared according to the reported methods [38,39,40,41,42,43]. First, 2-chloro-5-chloromethylpyridine was slowly added to the aqueous solution of NaHCO_3_ and 2-aminopyridine. Then, the mixture was refluxed for 5 h to afford intermediate **A**. Meanwhile, a simple formylation of 2-aminopyridine was created to give *N*-(pyridine-2-yl)formamide. Then, this compound underwent a nucleophilic substitution reaction along with 2-chloro-5-(chloromethyl)thiazole to produce intermediate **B**. Second, different substituted benzyl chlorides underwent at nucleophilic substitution reaction with diethyl malonate. Then, efficient alkaline hydrolysis was conducted under non-aqueous conditions by using dichloromethane/methanol (9:1) as solvent to provide 2-substituted malonic acid, and the mixture of 2-substituted malonic acid, 2,4,6-trichlorophenol, and phosphorus (V) oxychloride was refluxed for 3 h to obtain intermediate **C**. To the solution of intermediate **A** or **B** in toluene (1 mmol, 25 mL), intermediate **C** (1 mmol) was added and refluxed. The reaction was monitored by TLC. After completion of the reaction, the solvent was removed under reduced pressure, and the residue was purifed by column chromatography (ethyl acetate/methanol = 20/1) to give title compound **I**.

*3,4-dihydro-2,4-dioxo-1-((2-chloropyridin-5-yl)methyl)-3-benzyl-2H-pyrido[1,2-a]pyrimidin-1-ium-3-ide* (**I1**). Yellow solid; yield 44.7%; mp 65–67 °C. ^1^H-NMR (500 MHz, DMSO-*d*_6_) δ 9.23 (d, 1H, pyrido[1,2-*a*]pyrimidin Ar-H), 8.41 (s, 1H, 2-Cl-pyridin-5-yl 6-H), 8.20–8.19 (m, 1H, Ar-H), 7.73–7.70 (m, 2H, Ar-H), 7.47–7.44 (m, 2H, Ar-H), 7.31 (d, 2H, Ar-H), 7.20–7.18 (m, 2H, Ar-H), 7.17–7.08 (m, 1H, Ar-H), 5.53 (s, 2H, Ar-CH_2_-N), 3.78 (s, 2H, Ar-CH_2_-C). ^13^C-NMR (125 MHz, DMSO-*d*_6_) δ 159.6 (C=O), 154.3 (C=O), 149.7 (C=N, 2-Cl-pyridin-5-yl), 149.0 (Cl-C-N, 2-Cl-pyridin-5-yl), 146.3, 143.6, 142.3, 138.8, 131.9, 131.4, 128.7 (2C, Benzyl), 128.2 (2C, Benzyl), 125.7, 124.6, 116.8, 114.5, 92.8, 42.7 (CH_2_), 30.8 (CH_2_). ESI-HRMS (*m/z*): calculating for C_21_H_16_ClN_3_O_2_ [M + H]^+^ 378.1004, we obtained 378.0996.

### 4.2. Biological Assay

Bioassays of insecticidal activity against *A.* *craccivora* were investigated via a slightly modified FAO dip test method [44,45]. Tender shoots of soybeans with adult aphids were dipped in diluted solutions of the title compounds containing Triton X-100 (0.1%) for 5 s. Excess liquid was removed, and the shoots were placed in the conditioned room (25 ± 1 °C, 50% RH). Triflumezopyrim and Imidacloprid were used as positive controls. Mortality rates were recorded after 24 h.

### 4.3. Proteomics

#### 4.3.1. Sample Preparation

Tender shoots of soybeans with 50–100 adult *aphids* were dipped in 100 μg/mL of **I13** solution (diluted by Triton X-100) for 5 s. Excess liquid was removed, and the shoots were placed in the conditioned room (25 ± 1 °C, 50% RH). The control groups were handled with 0.1% Triton X-100, and each treatment was repeated thrice. Samples of control and **I13**-treated insects were collected at 12 h after treatment and were frozen for protein extraction [46].

#### 4.3.2. Proteins Extraction for LC−MS/MS Analysis

The total proteins of *A.* *craccivora* were extracted by a modified method [47,48]. First, samples of control and **I13**-treated insects were homogenized to fine powder (by mortar and pestle in liquid nitrogen). Ice-cold protein extraction buffer (0.5 M Tris-HCl (pH 7.5), 0.7 M sucrose, 0.1 M KCl, 50 mM EDTA, and 40 mM dithiothreitol (DTT)) lysed the total soluble protein at room temperature for 15 min. Then, after 30 min of shaking, extraction was conducted by an equal volume of Tris-phenol. Centrifugation was performed at 8000 g and 4 °C for 15 min (twice extraction). Five times volume of 0.1 M ammonium acetate in methanol was added to the collected supernatant, which was maintained at −20 °C overnight and then centrifuged at 8000 g for 10 min at 4 °C. Finally, the resulting pellets were washed by ice-cold acetone containing 1% (*w/v*) DTT thrice. After drying for 2 h in vacuum drier, the samples were dissolved in 100 μL of the rehydration solution (8 M (*w/v*) urea, 0.1 M (*w/v*) Tris, 10 mM dithiothreitol (DTT). Then, the concentration of total protein was determined using the Bradford method [49]. Before the LC-MS/MS analysis, protein was digested with trypsin using reported methods [50].

#### 4.3.3. LC-MS/MS Analysis, Database Searching, and Bioinformatics Analysis

All samples were analyzed via the LC-MS/MS combined system (Nano LC-1DTM plus system (Eksigent, Dublin, CA), TripleTOF 5600 MS (Foster City, CA, USA)). First, a full loop injection was used for 8 μL peptide samples. They were desalted on a ChromXP Trap column (Nano LC TRAP Column, 3 μm C18-CL, 120 A, 350 μm × 0.5 mm, Foster City, CA, USA). Then, the eluted samples were placed into column-Nano LC C18 reversed-phase column (3C18-CL, 75 μm × 15 cm, Foster City, CA, USA) for a second analysis. Under the flow rate of 300 nL/min, a combination of mobile phases, i.e., A mobile phase (5% ACN, 0.1% FA) and B mobile phase (95% ACN, 0.1% FA), was eluted over 120 min. Analyst (R) Software (TF1.6) can automatically switch between TOF–MS and Product Ion acquisition by the data-dependent mode on TripleTOF 5600 MS.

MaxQuant version 1.5.2.8 (http://www.coxdocs.org/doku.php?id=maxquant:common:download_and_installation)was used to manipulate raw data. The proteome of aphids was downloaded from UniProt, which contained 68,023 proteins that were searched via Andromeda search engine [51,52]. To ensure that only significant peptides were accepted for the identification, the false discovery rate (FDR) was set to 0.01. The difference of expression between the control group and treatment group was compared by the label-free quantification with a minimum of two ratio counts to determine the normalized protein intensity. The differentially accumulated proteins between control and treatment groups were identified via a two-sample unpaired *t*-test. The iBAQ value was used for *t*-test. Proteins with ANOVA analysis of *p* value ≤ 0.05 were considered differentially expressed.

All differentially expressed proteins were annotated with all aphid proteins using the DAVID 6.8 (https://david.ncifcrf.gov/content.jsp?file=Contact.html) [53,54]. The Fisher’s Exact Test (Fisher) exact test and FDR correction method [55,56,57] was used to identify the differentially expressed proteins based on GO (Gene Ontology, a gene function in a standardized classification system) categories in biological process (BP), cellular components (CC), and molecular functions (MF). The results are listed in Supplementary Materials Table S3. Some GO comments were listed after ranking according to the *p*-value sort. The smallest of the top 10 were shown in the column chart.

## 5. Conclusions

In summary, mesoionic pyrido[1,2-α]pyrimidinones derivatives containing a neonicotinoid moiety were designed, synthesized, and evaluated for their insecticidal activity. Results of bioassays indicated that these compounds displayed satisfactory insecticidal properties against *A. craccivora*. In particular, compound **I13** showed 92% mortality at a concentration of 100 μg/mL. Using the label-free proteomics to analyze the differentially expressed proteins after compound **I13** treatment, the differential expression of these proteins reflected the change in cellular structure and metabolism. Notably, these findings demonstrated that the synthesis of mesoionic pyrido[1,2-α]pyrimidinones derivatives containing a neonicotinoid moiety could be considered as a new template for pesticide development. These interesting bioactivities and responses of label-free quantitative proteomics led to further research by our group.

## Figures and Tables

**Table 1:** Insecticidal activities of the title compounds **I1–I28**.

Compounds	Concentration (μg/mL)	Mortality Rate (%)
**I1**	500	85
**I2**	500	69
**I3**	500	31
**I4**	500	23
**I5**	500	0
**I6**	500	51
**I7**	500	77
**I8**	500	51
**I9**	500	28
**I10**	500	46
**I11**	500	35
**I12**	500	43
**I13**	500	100
	200	100
	100	92
	50	30
**I14**	500	16
**I15**	500	0
**I16**	500	23
**I17**	500	49
**I18**	500	0
**I19**	500	58
**I20**	500	62
**I21**	500	39
**I22**	500	62
**I23**	500	58
**I24**	500	0
**I25**	500	62
**I26**	500	27
**I27**	500	46
**I28**	500	62
Imidacloprid	500	100
	200	100
	100	100
	50	100
Triflumezopyrim	500	100
	200	100
	100	100

## Supplementary Materials

The following are available online at http://www.mdpi.com/1420-3049/23/5/1217/s1, Table S1: identification of total protein, Table S2: identification of differentially expressed proteins, Table S3: results of differential protein GO analysis.
